# Wildlife Symbiotic Bacteria Are Indicators of the Health Status of the Host and Its Ecosystem

**DOI:** 10.1128/AEM.01385-21

**Published:** 2022-01-11

**Authors:** Maria Bravo, Theo Combes, Fernando O. Martinez, David Risco, Pilar Gonçalves, Waldo L. Garcia-Jimenez, Rosario Cerrato, Pedro Fernandez-Llario, Jorge Gutierrez-Merino

**Affiliations:** a Ingulados S.L., Cáceres, Spain; b School of Biosciences and Medicine, University of Surrey, Guildford, United Kingdom; c Neobeitar S.L., Cáceres, Spain; Unversidad de los Andes

**Keywords:** lactic acid bacteria, microbiota, One Health, symbionts, wildlife, immunomodulation

## Abstract

Lactic acid bacteria (LAB) are gut symbionts that can be used as a model to understand the host-microbiota cross talk under unpredictable environmental conditions, such as wildlife ecosystems. The aim of this study was to determine whether viable LAB can be informative of the health status of wild boar populations. We monitored the genotype and phenotype of LAB based on markers that included safety and phylogenetic origin, antibacterial activity, and immunomodulatory properties. A LAB profile dominated by lactobacilli appears to stimulate protective immune responses and relates to strains widely used as probiotics, resulting in a potentially healthy wildlife population, whereas microbiota overpopulated by enterococci was observed in a hostile environment. These enterococci were closely related to pathogenic strains that have developed mechanisms to evade innate immune systems, posing a potential risk for host health. Furthermore, our LAB isolates displayed antibacterial properties in a species-dependent manner. Nearly all of them were able to inhibit bacterial pathogens, raising the possibility of using them as an a la carte antibiotic alternative in the unexplored field of wildlife disease mitigation. Our study highlights that microbiological characterization of LAB is a useful indicator of wildlife health status and the ecological origin from which they derive.

**IMPORTANCE** The wildlife symbiotic microbiota is an important component for the greater diversity and functionality of their bacterial populations, influencing host health and adaptability to its ecosystem. Although many microbes are partly responsible for the development of multiple physiological processes, only certain bacterial groups, such as lactic acid bacteria (LAB), have the capacity to overpopulate the gut, promoting health (or disease) when specific genetic and environmental conditions are present. LAB have been exploited in many ways due to their probiotic properties, particularly lactobacilli; however, their relationship with wildlife gut-associated microbiota hosts remains to be elucidated. On the other hand, it is unclear whether LAB such as enterococci, which have been associated with detrimental health effects, could lead to disease. These important questions have not been properly considered in the field of wildlife and, therefore, should be clearly addressed.

## INTRODUCTION

Symbiont microbes play an essential role in the natural defense against infectious diseases (1, 2). The host’s immune system recognizes microbial ligands, known as microbial-associated molecular patterns (MAMPs), through pattern recognition receptors (PRRs), thereby distinguishing between resident and pathogenic bacteria (2). MAMPs include a wide variety of molecules and structural components, such as exopolysaccharides (EPS), membrane lipoproteins, lipoteichoic and wall teichoic acids (LTA and WTA, respectively), pili, and fimbriae (2, 3). These MAMPs can be recognized by PRRs such as Toll-like receptors (TLRs), resulting in the activation of protective signaling pathways that are mediated by several transcription factors, including the nuclear factor-kappa B (NF-κB) and interferon regulatory factors (IRFs) (4, 5). Furthermore, some bacteria contribute to host health by competing and inhibiting a broad spectrum of pathogens via the production of antimicrobial substances, including fermentation metabolites such as lactic acid and ethanol, as well as antimicrobial peptides like bacteriocins (6–8). Although many resident bacteria are responsible for the development of beneficial physiological processes, others, known as pathobionts, could promote disease when specific genetic or environmental conditions are altered in the host (9). In this respect, the microbes of wild animals are not an exemption, as they may exert beneficial or detrimental properties, all specifically related to the activation of metabolic and immunomodulatory pathways (7, 10, 11). In fact, the wildlife gut microbiota is more diverse than that of its domestic homologues (12, 13), influencing significantly the health of the host and its adaptability to the environment (14).

The number of studies addressing the natural and symbiotic interaction of the host and its microbiota in wildlife populations is very limited (6). Only a few authors have explored the potential use of some prototypical symbionts, such as lactic acid bacteria (LAB), as an environmentally friendly and sustainable alternative to prevent disease transmission and dissemination in wildlife (3, 15). Certain LAB genera, such as *Lactobacillus*, have been exploited in many ways due to their probiotic properties and their capacity to enhance physiological processes. However, other LAB genera, such as *Enterococcus*, may act as pathobionts, triggering infections and subsequent pathological disorders (16–18). Therefore, it is important to determine whether LAB behave as a beneficial commensal or pathobiont, especially in the context of wildlife, where monitoring the whole microbiome can be a difficult task. Some microbiological markers such as virulence factors or antibiotic resistance genes can be very useful indicators of host health (9, 19), and the phylogenetic origin of bacteria may provide valuable information about their ecological evolution and physiological relationship with the host (20, 21). LAB are an excellent model to understand host-microbiota cross talk in wildlife (19, 22).

In our previous work (23), we isolated LAB from three wild boar populations that shared the common characteristic of living in estates free of tuberculosis (TB) despite being surrounded by areas with significant clinical history of TB affecting both domestic and wild species. These populations are also offered with nutritional supplements under controlled conditions. Our findings showed that lactobacilli were the predominant LAB population found in fecal samples and that these bacteria antagonized Mycobacterium bovis, the causative agent of TB. Here, we have implemented the same experimental protocol but including fecal samples from wild boar populations located in a different environment and with a high TB prevalence, in contrast to the estates of our previous study. In this new scenario, animals live on a reserve of land with minimal human contact, and the TB seroprevalence is much higher, with animals suffering from significant gross lesions. We hypothesize that resident LAB contribute to maintain (or perpetuate) infectious diseases in wildlife and that the LAB profile is influenced by the ecosystem where wild boar populations are located.

The aim of this study was to determine whether the phenotype and genome of typical gut symbionts such as LAB can provide the health status of a wildlife ecosystem. We isolated viable LAB from wild boar populations with different TB epidemiological situations, and those isolates representing the most abundant LAB genera in each of the populations were subjected to a very comprehensive phenotypic and genotypic characterization. This characterization was carried out using the following 3 LAB markers: (i) safety and origin, (ii) antibacterial activity, and (iii) immunomodulatory properties. The phenotypic examination included (i) sensitivity (tolerance) to antibiotics, (ii) antimicrobial activity against wild boar pathogens, and (iii) phagocytic intake and ability to activate NK-kB and IRF pathways in phagocytes. The resulting phenotypes were then complemented with a genome analysis that comprised the identification of (i) antimicrobial resistance determinants, virulence factors, and phylogenetic proximity to other LAB; (ii) bacteriocin clusters and additional genes encoding antimicrobial metabolites; and (iii) immunomodulatory molecules.

## RESULTS

### Wild boar with opposite TB epidemiologies harbor different LAB.

We isolated 30 LAB colonies from wild boar displaying antimicrobial activity against two indicator strains, M. smegmatis and M. luteus, that have proved to be very reliable and informative (3, 23). M. luteus is very sensitive to a broad range of different antimicrobial compounds, while M. smegmatis is a fast-growing Mycobacterium species that facilitates rapid anti mycobacterial screening. The LAB isolates were obtained from animals representing 4 different states with different TB epidemiologies, and the number of isolates per state varied between 5 and 10. Following bacterial species identification by Gram staining, catalase/oxidase tests, and 16S rRNA sequencing, two different LAB profiles were clearly observed. Nearly all isolates from estates 1, 2, and 3 (group 1, zero TB prevalence) were identified as *Lactobacillus* sp., whereas estate 4 (group 2, high TB prevalence) showed a very predominant *Enterococcus* sp. profile. A small number of *Pediococcus* sp. isolates were found in both groups. Subsequently, we selected a total of 11 LAB representing not only each of the estates but also different species and strains. The LAB isolated included *L. plantarum* (C1), Lactobacillus salivarius (C2 and C12), and Pediococcus acidilactici (C5) from estate 1; *L. plantarum* (EML1) from estate 2; Lactobacillus plantarum (SA3) and Lactobacillus paracasei (SA5) from estate 3; and Pediococcus acidilactici (R91), Enterococcus faecalis (A1 and R8), and Enterococcus casseliflavus (R95) from estate 4. All species were confirmed by whole-genome sequencing.

### The safety profile of LAB depends on the strains and species.

**FIG 1:**
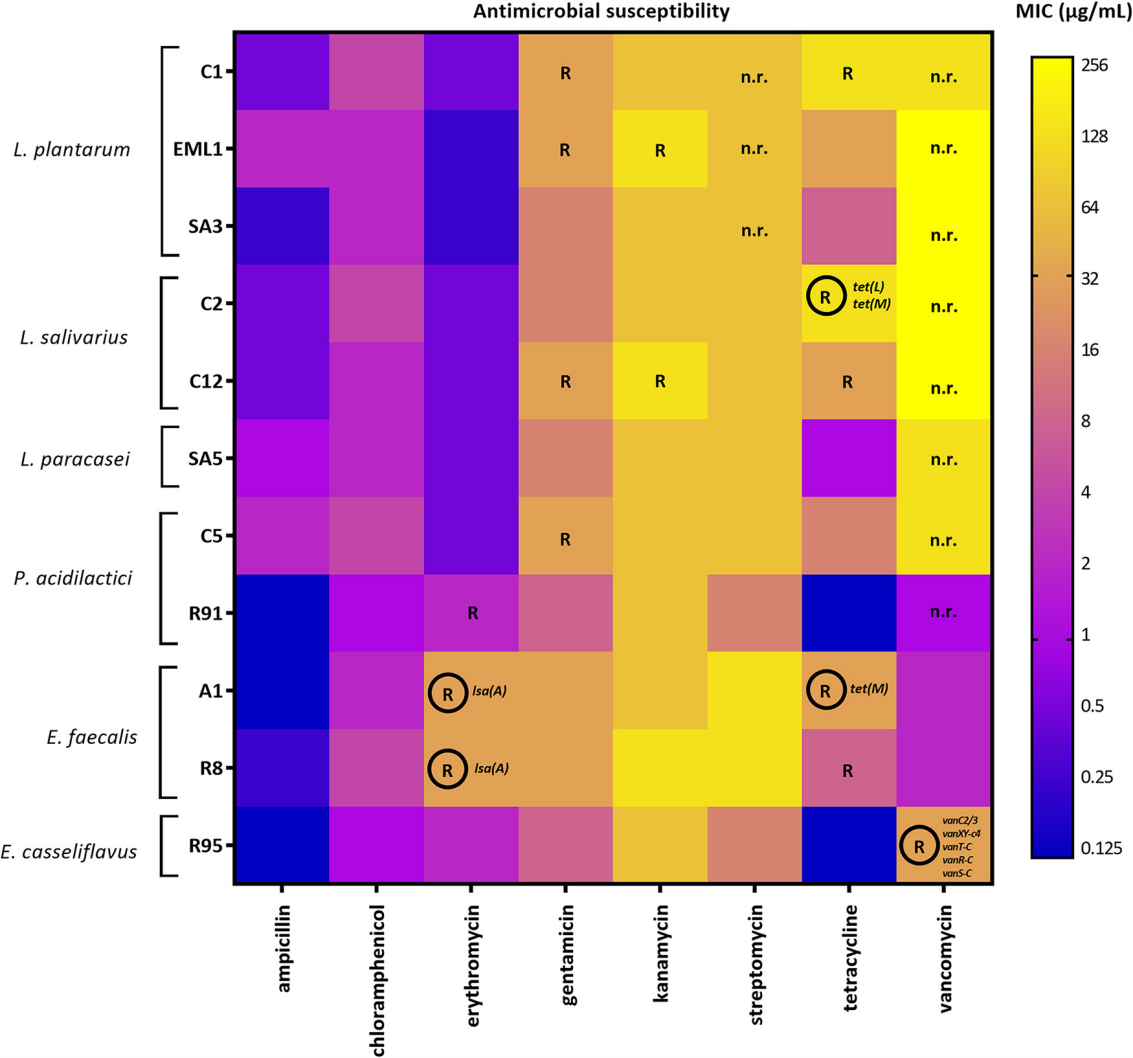
Heatmap showing the susceptibility of LAB isolates to antibiotics frequently used in veterinary and human medicine. Susceptibility was quantified using MICs (mg/liter) and is represented as a color gradient. LAB strains that are recognized as resistant by the FEEDAP standards are indicated as R. Rounded R indicates agreement between genotypic and phenotypic resistance, with the resistance gene markers shown in italics. Assessment of vancomycin and streptomycin is not required (n.r.) for lactobacilli and pediococci due to their innate natural resistance to these antibiotics.

To assess the safety of our LAB isolates, we first quantified their susceptibility to antibiotics that are commonly used in human and veterinary medicine (Fig. 1). Simultaneously, we searched for genes associated with antimicrobial resistance (AMR) to correlate these resistance markers with the phenotypic tolerance of the isolates (Fig. 1). All isolates were susceptible to ampicillin and chloramphenicol. With the exception of E. faecalis, LAB were also sensitive to erythromycin. The genomes of the E. faecalis strains revealed the presence of *lsa*(A), a gene encoding an efflux pump that export macrolides out of the cell. Interestingly, nearly all LAB isolates were relatively tolerant to aminoglycosides (gentamicin, kanamycin, and streptomycin), and some of them, strains of *L. plantarum*, *L. salivarius*, and *P. acidilactici*, were identified as resistant by the FEEDAP standards. However, no AMR markers were located in any of their genomes. On the contrary, we identified markers associated with tetracycline resistance in strains of *L. salivarius* and E. faecalis, *tet*(L) and *tet*(M). Together with *L. plantarum*, these two species showed tolerance to tetracycline. Furthermore, we observed that *E. casseliflavus* tolerates vancomycin and that this phenotypic feature correlates with the presence of multiple vancomycin resistance genes (*vanC2-3*, *vanXY-c4*, *vanT-C*, *vanR-C*, and *vanS-C*). Interestingly, none of the *P. acidilactici* strains carry markers associated with AMR.

**FIG 2:**
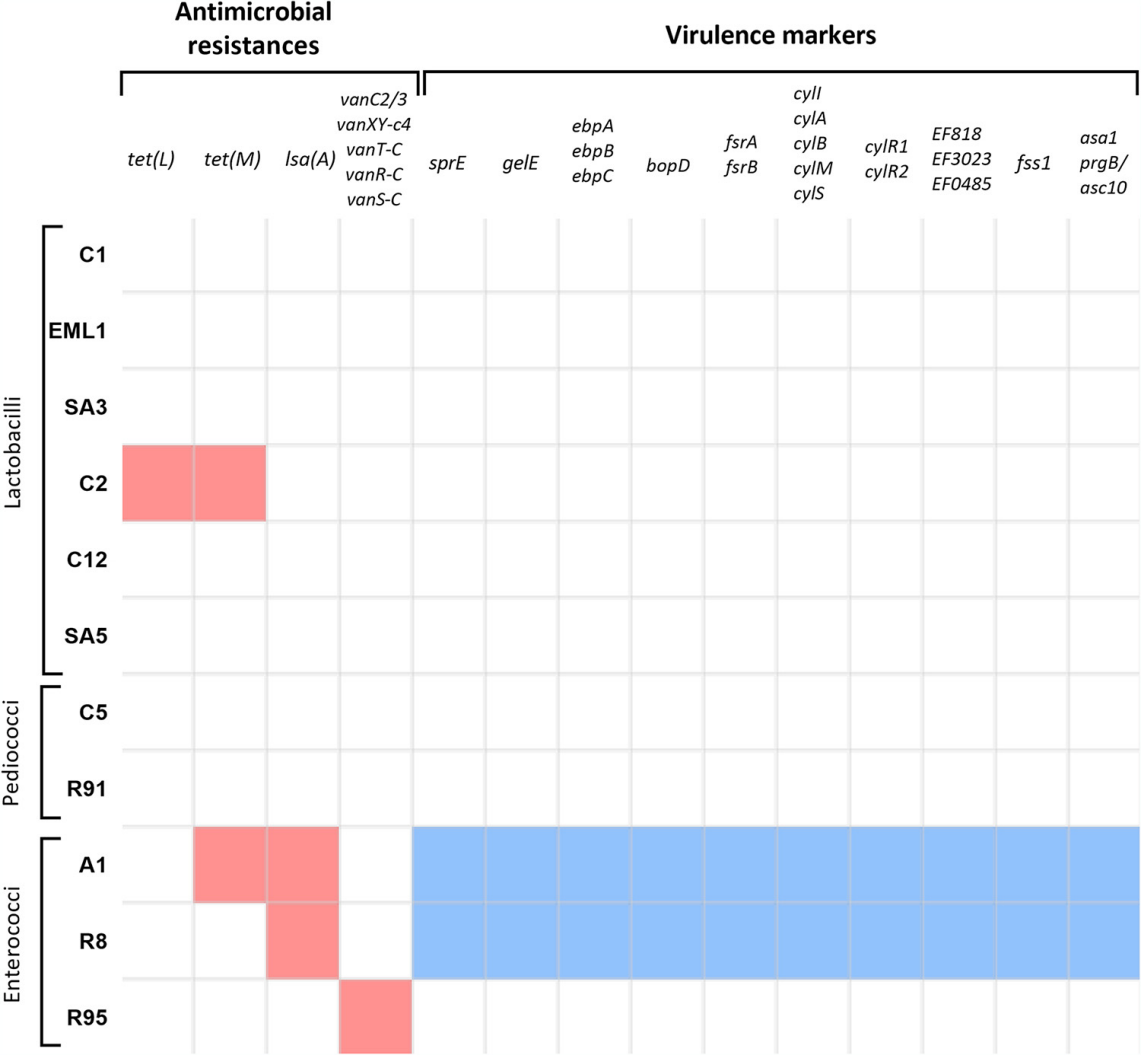
Antimicrobial resistance genes and genetic virulence markers. Except for *L. salivarius* C2, which has genes for resistance to tetracyclines, lactobacillus and pediococcus isolates lack resistance and virulence genes. Enterococcal isolates present numerous genetic markers of antimicrobial resistance and virulence, especially the two isolates of E. faecalis.

To complement the safety profile of the LAB isolates, we also searched for virulence markers in their genomes. We found that the two E. faecalis strains possess several virulence genes, including a serine protease (*sprE*), a gelatinase (*gelE*), a biofilm formation regulator (*bopD*), virulence system regulators (*fsrA* and *fsrB*), and hyaluronidases (*EF0818*, *EF3023*, and *EF0485*). The genomes of lactobacilli and pediococci showed no virulence factors (Fig. 2).

### The phylogenetic proximity of LAB varies between strains isolated from food, animals, and humans.

To determine the phylogenetic background of our 11 LAB isolates, we conducted a core genome single-nucleotide polymorphism (SNP) analysis using NCBI publicly available genomes. We observed that the *L. plantarum* isolates EML1 and SA3 are almost identical and that both show similarities with isolate C1 (see Fig. S1 in the supplemental material). All of them seem to be related to strains isolated from fermented food or probiotic-related products. Similarly, *L. paracasei* SA5 locates close to strains derived from food, in particular dairy products manufactured for human consumption (Fig. S2). The *L. salivarius* isolates C2 and C12 clustered between two other groups representing strains from healthy pigs and humans (Fig. S3). Interestingly, the *P. acidilactici* isolates C5 and R91 showed a close proximity to strains of diverse origin, including food, animals, and humans (Fig. S4). However, a significant divergence was observed between these two pediococci. Isolate C5 belongs to a group of other strains isolated from fermented food, humans, and pet animals (dog), whereas R91 relates to a different group of strains that derive from food (fermented and dairy) and farm animals (chicken and pig). Furthermore, both E. faecalis isolates (A1 and R8) are closely related to pathogenic strains isolated from pigs (Fig. S5). *E. casseliflavus* isolates were closely related to other pathogenic isolates but derived from human patients.

### LAB from TB-free wild boar outcompete BCG and encode unmodified bacteriocins and a significant number of H_2_O_2_-producing enzymes.

**FIG 3:**
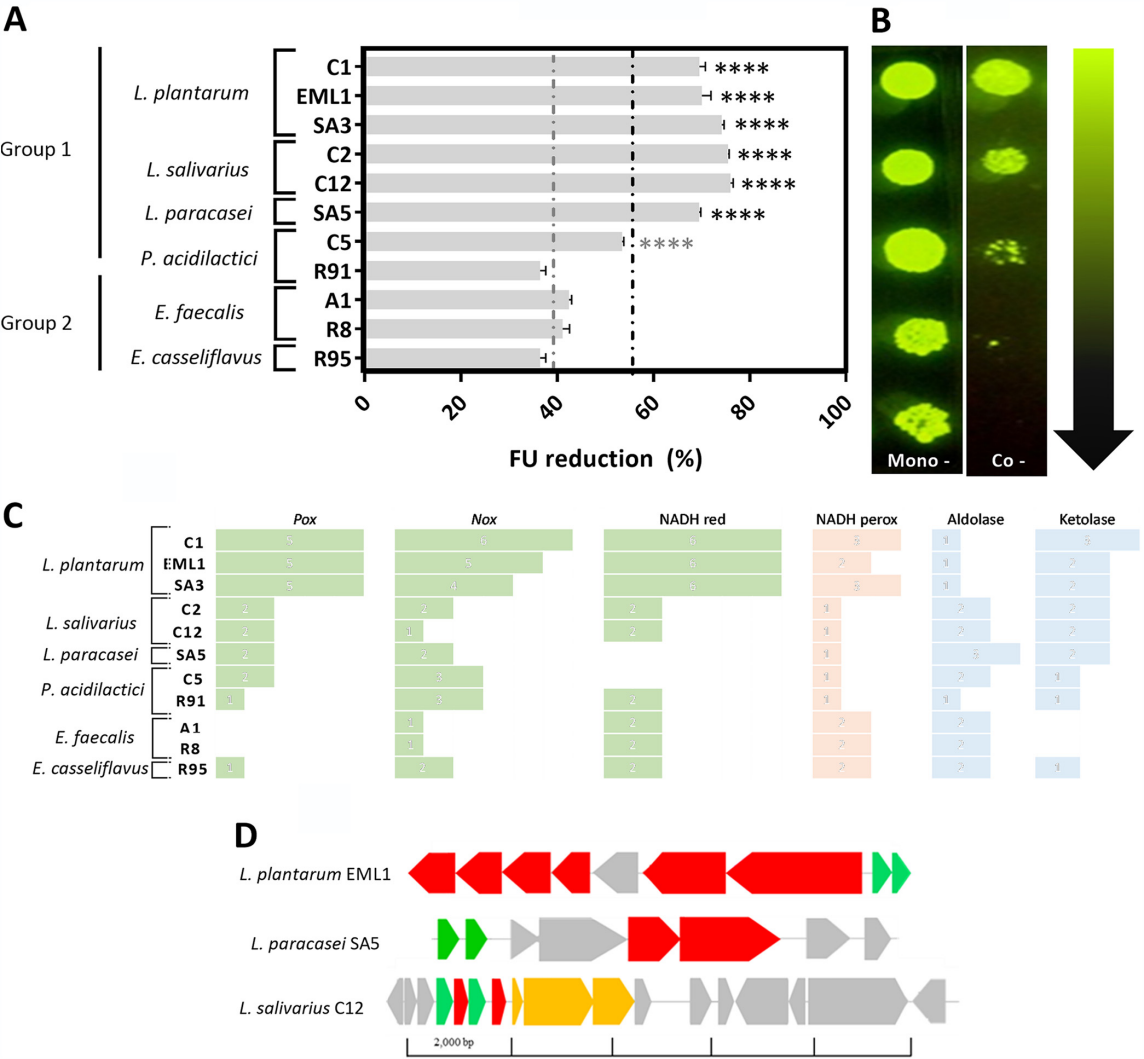
LAB isolated from TB-free wild boar show a strong antimycobacterial activity and potentially produce unmodified two-peptide bacteriocins and a large variety of antimicrobial secondary metabolites. (A) Reduction in fluorescent units (FU) from cocultures of M. bovis BCG-GFP with LAB after 48 h of incubation. FU reduction was calculated using at least 3 biological replicates and normalized with the FU of BCG monocultures. (B) Mycobacterial selective agar containing CFU of BCG-GFP from a monoculture or a coculture with an *L. plantarum* isolate. The serial log dilutions obtained from the coculture show a very significant reduction in CFU (data not shown). (C) Genes associated with the production and accumulation of H_2_O_2_ (green and orange) and other secondary metabolites, such as lactate, acetate, ethanol, and CO_2_ (blue), in the LAB isolates. (D) Gene clusters involved in the biosynthesis of unmodified two-peptide bacteriocins in *L. plantarum*, *L. paracasei*, and *L. salivarius*. Genes encode precursor bacteriocins (green), transport/immunity proteins (red), regulatory proteins (yellow), and other hypothetical proteins (gray).

The 11 selected LAB were cultured simultaneously with 4 indicator pathogenic/clinical isolates: GFP-BCG, Escherichia coli, Pasteurella multocida serotype B, and Listeria monocytogenes. We first observed a significant antimicrobial effect against the BCG isolate (Fig. 3A and B). All lactobacilli from group 1 (TB-free states) reduced significantly the green fluorescent protein (GFP) emission of BCG, and this reduction positively correlated with BCG counts, as reported in our previous work (23). Conversely, all enterococci from group 2 (TB state) showed a much more limited antimycobacterial effect, with a reduction that was 2-fold lower than that observed with lactobacilli. Remarkably, this antimicrobial difference between isolates of lactobacilli and enterococci was also recorded with the two isolates of *P. acidilactici*. C5 from TB-free group 1 displayed a stronger antimicrobial activity against BCG than with R91 from TB group.

As an attempt to correlate the antimycobacterial effect of the LAB isolates with their genotype, we searched for antibacterial compounds in the genomes. All *Lactobacillus* species carry gene clusters involved in the biosynthesis of unmodified two-peptide bacteriocins (Fig. 3D) and a very prolific set of genes associated with the production of H_2_O_2_, in particular *L. plantarum* (Fig. 3C). Interestingly, the number of H_2_O_2_ markers decreases progressively within the pediococci and enterococci. Important H_2_O_2_-producing genes such as *pox* and *nox* are absent in E. faecalis, and although they remain present in *P. acidilactici* and *E. casseliflavus*, this occurs at different *pox*-*nox* ratios (2:3 in C5; 1:3 in R91; and 1:3 in R95). Furthermore, we observed that all lactobacilli contain fructose-6-phosphate aldolase (Embden-Meyerhof pathway [EMP] pathway) and at least two genes encoding phosphoketolase (PKP) (Fig. 3C), demonstrating their ability to simultaneously produce lactate, acetate, ethanol, and carbon dioxide. The *Pediococcus* isolates and *E. casseliflavus* are also facultative heterofermenters, but their genomes only harbor one PKP gene. Conversely, the two E. faecalis strains act as obligate homofermenters, as their genomes only possess the fructose-6-phosphate aldolase gene, converting sugars into lactate via EMP.

### The antimicrobial activity that LAB show against clinical isolates is species specific.

**FIG 4:**
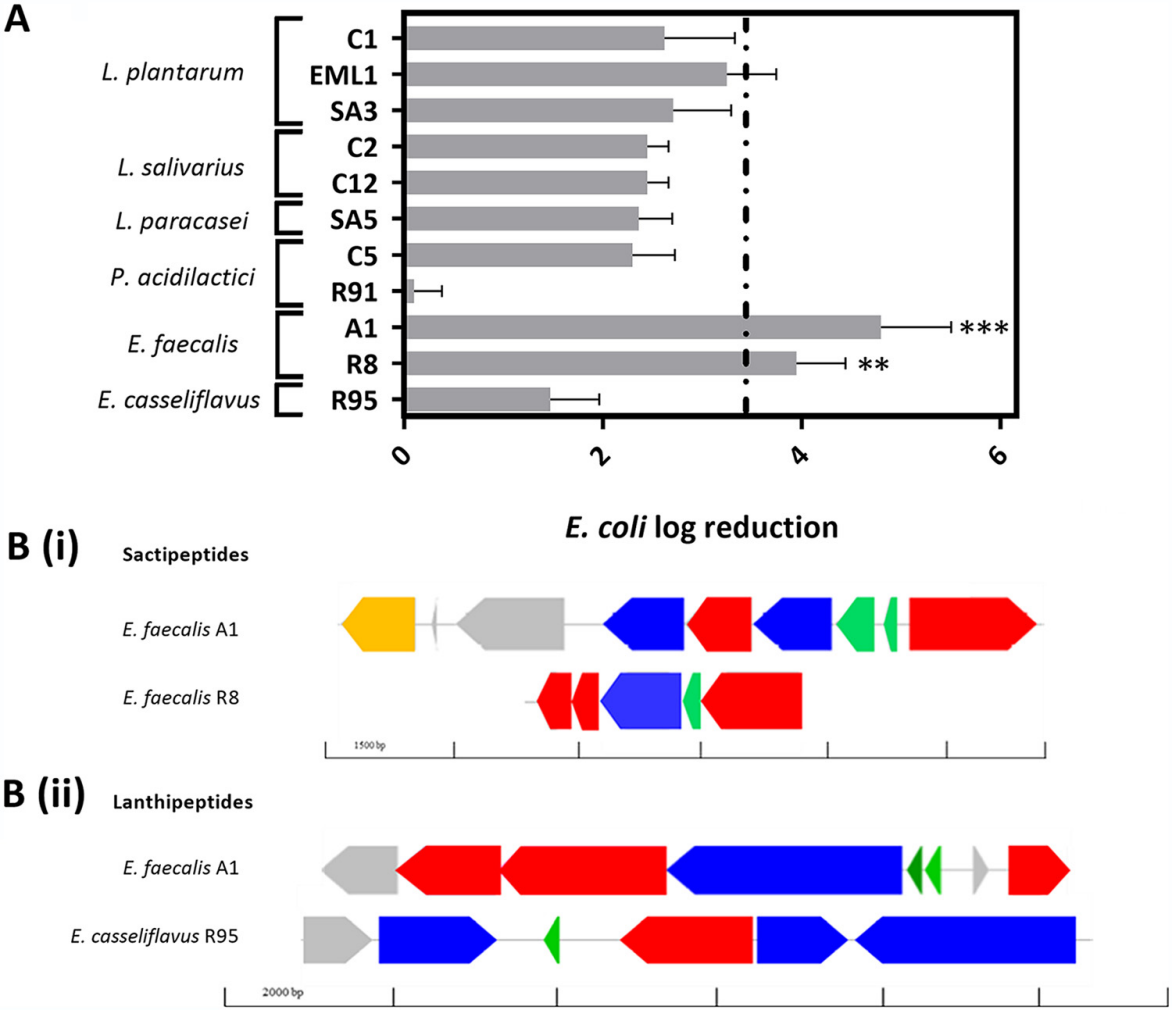
E. faecalis inhibits E. coli and potentially produces posttranslationally modified bacteriocins. (A) Log reduction in number of CFU/ml of E. coli when cocultured with LAB after 24 h of incubation. Log reduction was calculated using at least 3 biological replicates and then normalized with the number of CFU/ml of E. coli monocultures. (B) Gene clusters involved in the biosynthesis of sactipeptides (i) and lanthipeptides (ii), two posttranslationally modified bacteriocins found in the E. faecalis isolates A1 and R8 and *E. casseliflavus* R95. The different colors for the genes represent precursor bacteriocins (green), posttranslational modification enzymes (blue), transport/immunity proteins (red), and other hypothetical proteins (gray).

**FIG 5:**
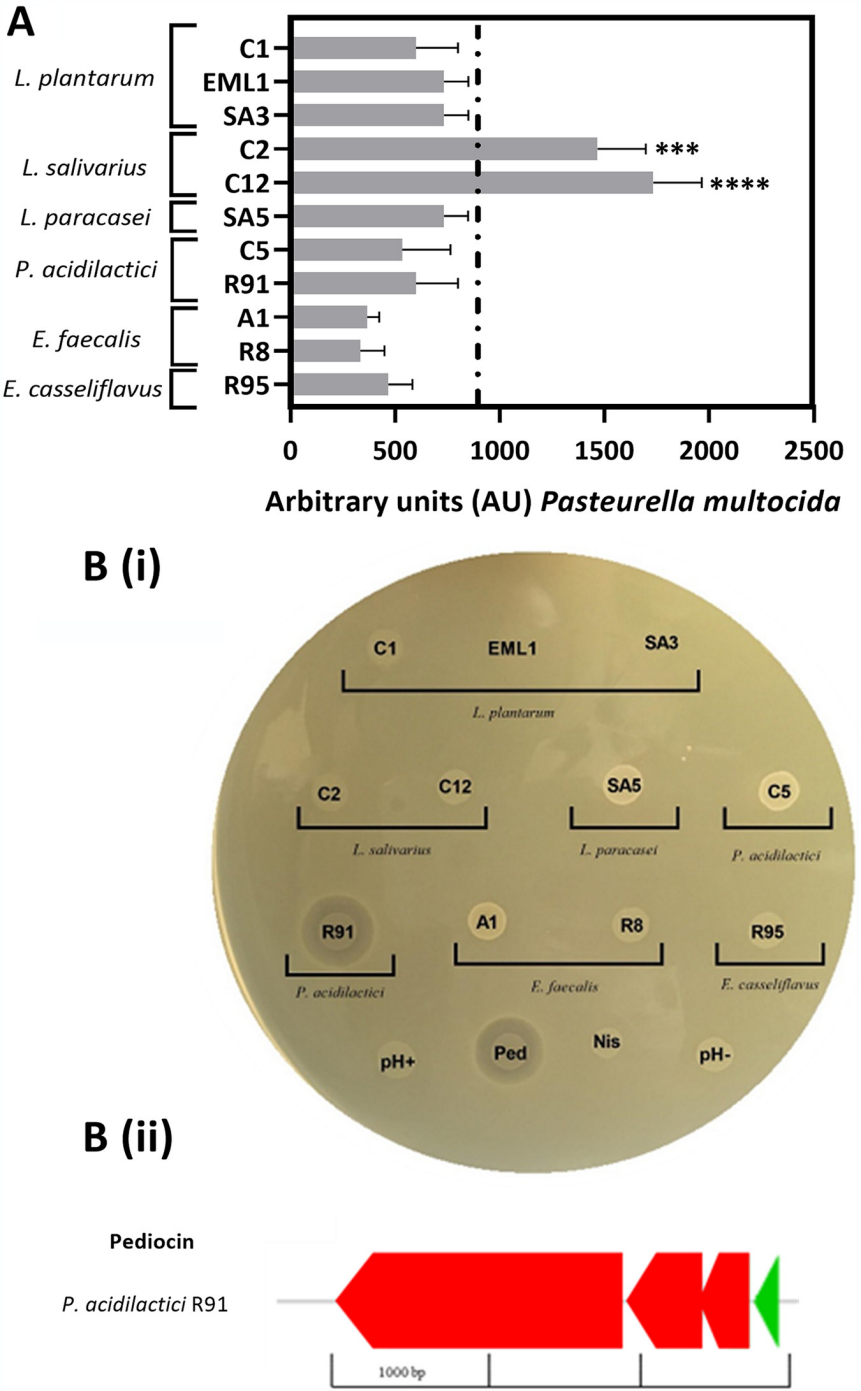
Extracellular activity displayed by *L. salivarius* and *P. acidilactici* against Gram-positive or -negative pathogens. (A) Arbitrary units per milliliter (AU/ml) of supernatants obtained from LAB against P. multocida serotype B after an overnight incubation at 37°C. AU/ml is defined as the reciprocal of the greatest dilution showing clear growth inhibition of the pathogen. (B) *P. acidilactici* R91 shows a clear antilisterial activity and potentially produces unmodified single-peptide bacteriocins. (i) Spot-on-agar test of LAB cultures against Listeria monocytogenes. Cultures of *L. plantarum* WCFS-1 (pH+, pH acidifier), *P. acidilactici* PA1.0 (pediocin producer, Ped), Lactococcus lactis NZ9700 (lanthipeptide nisin producer, Nis), and Lactococcus lactis NZ9800 (pH-, moderate pH acidifier) were used as controls. (ii) Gene cluster involved in the hypothetical synthesis of pediocin PA-1 in Pediococcus acidilactici R91.

Among all the antimicrobial methods used, we found that cocultures were very useful to observe significant differences in the antibacterial activity that our LAB isolates display against E. coli. With regard to P. multocida and L. monocytogenes, the broth microdilution assay and spot-on-agar test results were much more informative than the cocultures. Additionally, we employed the same genome procedure as that indicated above for the anti-BCG screening to look into antimicrobial markers. On the one hand, we observed that the two E. faecalis strains show the highest antimicrobial activity against E. coli (Fig. 4A) and that both contain gene clusters involved in the biosynthesis of sactipeptides (Fig. 4Bi), a group of posttranslationally modified bacteriocins characterized by the presence of linkages between the sulfur of cysteines and the alpha carbon of other residues. Additionally, the E. faecalis isolate A1 and *E. casseliflavus* R91 carry genes coding for a lanthipeptide (Fig. 4Bii), another bacteriocin that is modified by posttranslational thioether bonds (24). On the other hand, supernatants from *L. salivarius* cultures displayed a very strong activity against P. multocida (Fig. 5A), and one *P. acidilactici* isolate (R91) showed a clear halo of inhibition against L. monocytogenes (Fig. 5Bi). BAGEL 4 genome analysis revealed that the two *L. salivarius* isolates (C2 and C12) harbor a considerable number of genes encoding bacteriolysins and that R91 possesses a gene cluster associated with the production of pediocin (Fig. 5Bii).

### LAB isolated from TB-free wild boar interact with phagocytes and activate innate immune responses.

**FIG 6:**
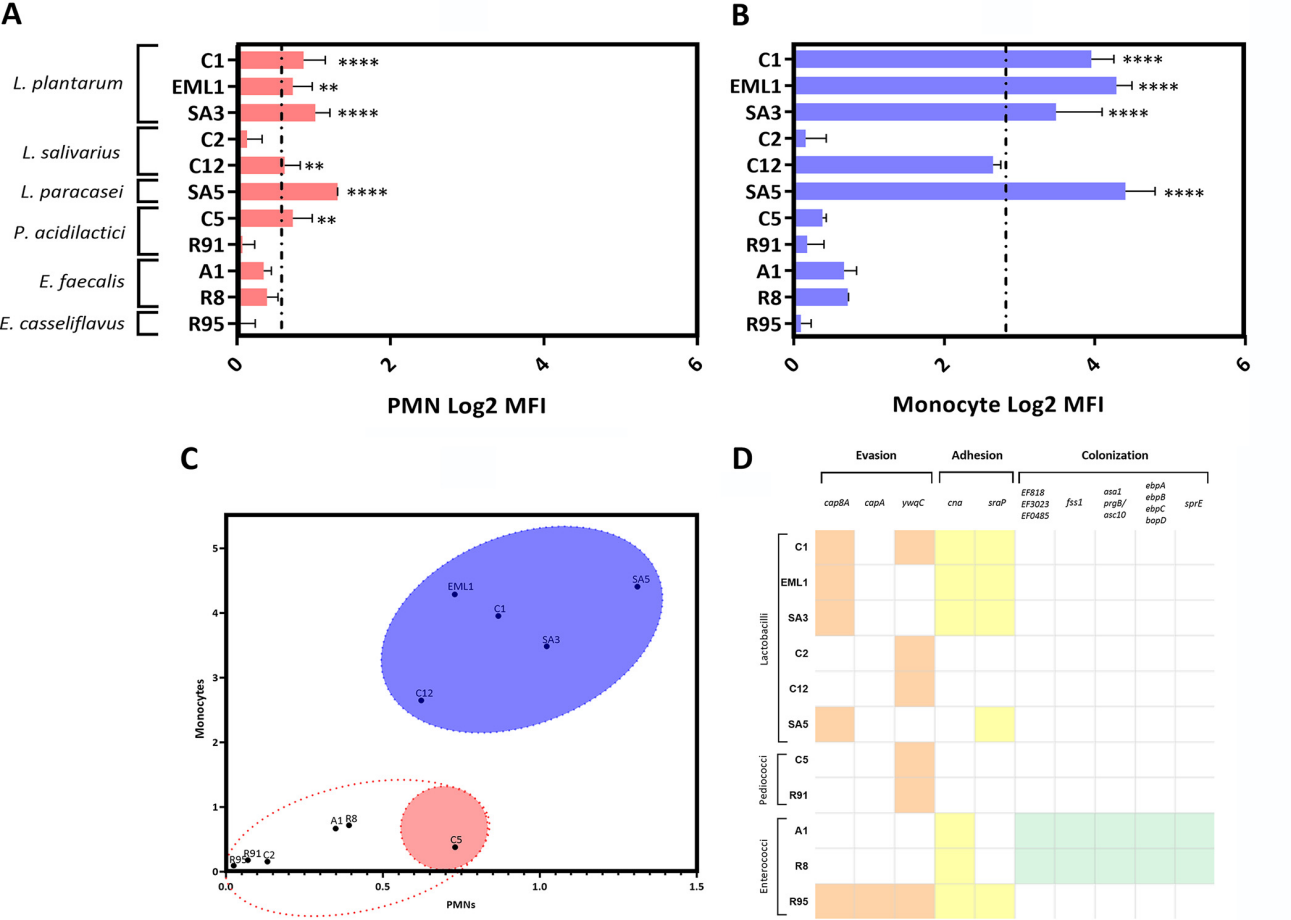
Lactobacilli interact with porcine phagocytes and carry molecules associated with cellular adhesion. The response of PMNs (e.g., neutrophils) (A) and monocytes (B) to LAB was quantified using PKH2-labeled bacteria and log_2_ mean fluorescence intensity (MFI). MFI was normalized with a blood control and the phagocytes derived from 2 healthy pig donors. (C) The phagocytic profile of most *Lactobacillus* isolates is predominantly monocytic, but *L. paracasei* also interacts with PMNs. Enterococci and pediococci show a very poor interaction with phagocytes. (D) LAB isolates carry genes that associate with evasion, adhesion, and/or colonization.

**FIG 7:**
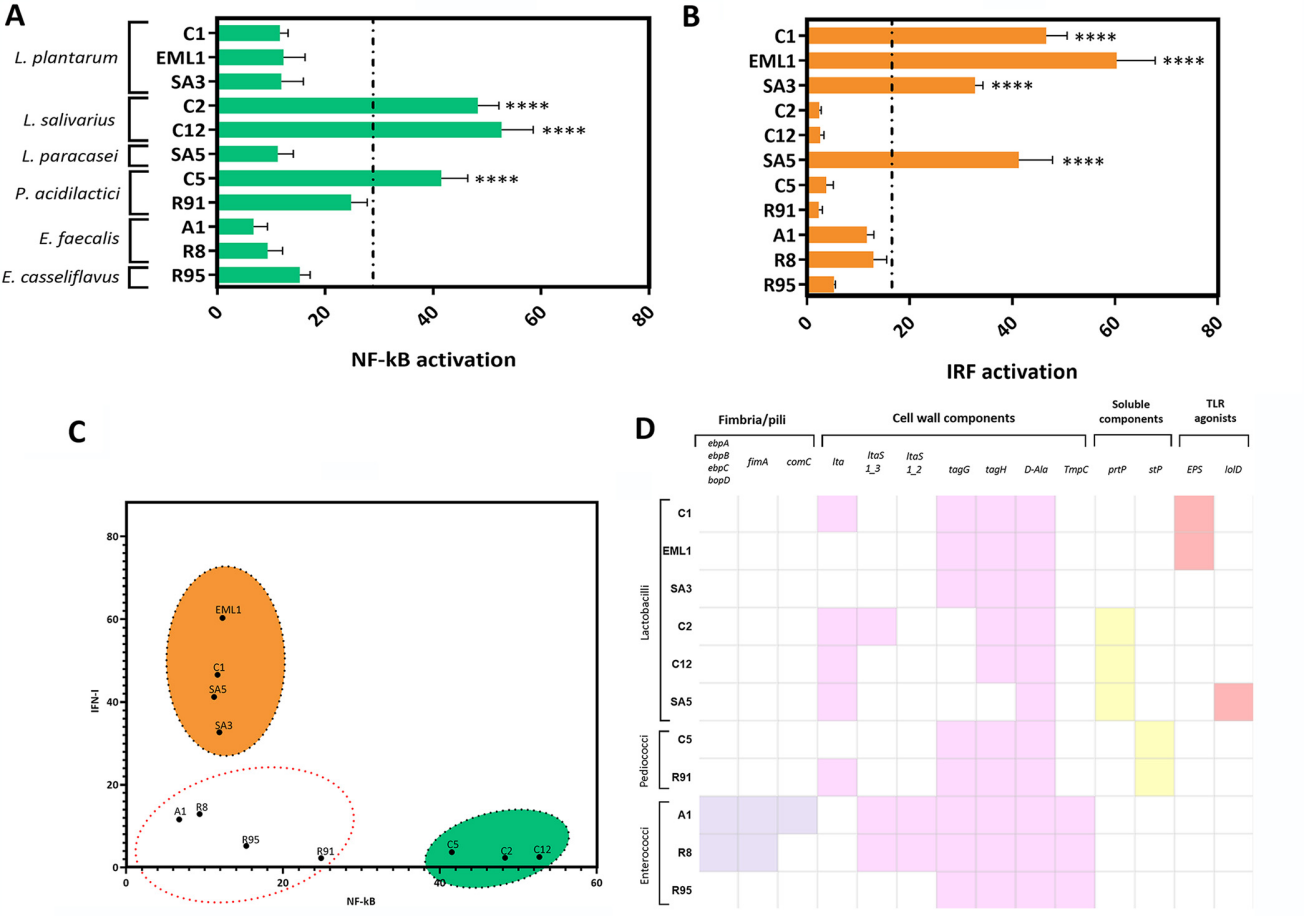
Lactobacilli activate protective innate immune responses in macrophages and possess molecules associated with TLR activation. Activation of NF-κB (A) and IRF (B) was quantified in PMA-differentiated THP-1 macrophages exposed to LAB at a ratio of 20 bacteria per macrophage. The NF-κB/IRF activation is presented as fold increase over a nonstimulated condition, and data represent at least 3 biological replicates. (C) LAB isolates distribute differently based on their ability to activate NF-κB/IRF pathways. The *Lactobacillus* species *L. plantarum* and *L. paracasei* trigger significant IRF responses, whereas *L. salivarius* predominantly induces NF-κB activation. (D) LAB isolates possess genes associated with pattern recognition receptors (PRRs), such as TLRs and NOD-like receptors, including fimbria/pili, cell wall components, and TLR2 agonists.

The phagocytic profile of the LAB isolates was first determined by fluorescence-activated cell sorting (FACS) using porcine blood and PKH2-labeled bacteria (Fig. 6). With the exception of *L. salivarius* C2, all lactobacilli strongly interacted with phagocytes (Fig. 6A and B) and monocytes were much more responsive than neutrophils (Fig. 6C). However, *L. paracasei* also interacted with neutrophils. None of the *Pediococcus* or *Enterococcus* isolates showed any significant interaction with phagocytes. Remarkably, the phagocytic profile of lactobacilli positively correlated with their ability to activate protective innate immune responses in macrophages. All lactobacilli induced the activation of NF-κB or IRF in a species-dependent manner (Fig. 7A and B). Both *L. plantarum* and *L. paracasei* trigger a very significant IRF activation, whereas *L. salivarius* induces a potent NF-κB activation (Fig. 7C). The *P. acidilactici* isolate from TB-free group 1 (C5) also activated a significant NF-κB response. All isolates that derive from TB group 2 (enterococci and *P. acidilactici* R91) did not activate either of the pathways (Fig. 7A to C).

As a complementary investigation, we used the genomes of the isolates to identify markers associated with innate immune responses. On the one hand, we found genes encoding proteins that have been reported to be involved in mechanisms of evasion, adhesion, and colonization (Fig. 6D). A significant number of adhesion proteins were found in lactobacilli, especially in *L. plantarum* and *L. paracasei*, while the enterococcus isolates have the capacity to express proteins related to evasion and colonization. On the other hand, the genome annotations gave us additional molecules that associate with the activation of PRRs (Fig. 7D). We identified not only conventional PRR ligands such as EPS, pili, LTA, WTA, and lipoproteins but also enzymes and serine/threonine (S/T)-rich proteins (prtP and stP) that convert proteins into immunomodulatory oligopeptides or with capacity to stimulate immune cells (2, 25).

## DISCUSSION

Commensal bacteria live in symbiosis with animals, but their relationship can change under different environmental and physiological conditions (9). While some of these bacteria can protect hosts and exert health-promoting functions (17), others may trigger infections and jeopardize host health (26). In particular, certain populations of gut commensals may influence health or disease outcome, reflecting the lifestyle of their hosts (27). It is evident that a better understanding of the origin of commensal bacteria and how these interact with pathogenic bacteria and animal cells is critical to determine the health status of the host and its surrounding ecosystem. In this study, we have examined biologically relevant responses and genome sequencing from typical gut residents such as LAB that were isolated from two different wildlife ecosystems. The first ecosystem comprised wild boar populations with zero prevalence of TB despite being surrounded by areas with significant clinical history of the disease (28). This group showed an LAB profile dominated by lactobacilli. On the contrary, the main LAB observed in the second ecosystem, TB high-risk area, was predominantly enterococci. Pediococci were found in both ecosystems. Based on these initial observations, we proceeded to evaluate whether opposed LAB profiles correlate with different wild boar ecosystems.

First, we observed that the enterococci of the wild boar group 2 (high TB prevalence) carry a significant number of AMR genes and virulence factors. We know that bacteria such as *Enterococcus* species adapt to hostile environments because of their plasticity to develop or acquire mechanisms of resistance and persistence (29). Some of these mechanisms include the horizontal transmission of genes associated with virulence and AMR. Although AMR is not considered a virulence factor, both virulence and antibiotic resistance contribute to the ability of *Enterococcus* to survive (30). As previously described in wild boar (31), the E. faecalis strains A1 and R8 are resistant to trimethoprim and erythromycin and carry genes, *dfrE* and *lsa*(A), that confer resistance to these two antibiotics (32). Tetracycline tolerance was also displayed by the two E. faecalis strains, but only strain A1 contains a tetracycline resistance gene, *tet*(M), that is frequently found in plasmids (33, 34). Furthermore, the species *E. casseliflavus* showed a significant resistance to vancomycin, and this phenotype strongly correlated with the genotype of the strain. Several determinants associated with vancomycin resistance (35, 36) were identified in its genome. On the other hand, the genomes of the two E. faecalis isolates revealed the presence of virulence regulators that seem to play an important role in pathogenesis (37, 38) as well as additional proteins that have extensively been described as virulence factors (38–41). These proteins included proteases, gelatinases, hyaluronidases, cytolysins, aggregation substances, capsular polysaccharide, and surface- and biofilm-associated proteins.

In contrast to enterococci, isolates of lactobacilli and pediococci did not carry virulence factors and were susceptible to some of the antibiotics tested in this study. In fact, most of the strains and species of *Lactobacillus* and *Pediococcus* are used in probiotic formulations due to their qualified presumption of safety (QPS) status (42). However, a full safety assessment is suggested to ensure their security, particularly for acquired antimicrobial resistance (43). We found that our lactobacilli and pediococci have no acquired AMR genes and that the tolerance displayed against vancomycin, gentamicin, kanamycin, streptomycin, and tetracycline is innate, as previously reported (44–50). Only one strain of *Lactobacillus* (*L. salivarius* C2) carries the tetracycline genes *tet*(L) and *tet*(M), the most widespread acquired resistance determinants found in lactobacilli (46, 50). Our data confirm that the presence of AMR genes is more prevalent in enterococci than in other LAB such as lactobacilli or pediococci (33).

To investigate the origin of the LAB isolates and their relationship with the host and its ecosystem, we generated phylogenetic trees using the genomes of our strains and other closely related strains isolated from different sources (51). We found that *L. plantarum* and *L. paracasei* are mainly related to fermented food and dairy products, whereas the two *L. salivarius* strains associate with other strains isolated from healthy animals. These observations are in agreement with previous studies that propose that the lifestyle of *L. salivarius* is more adapted to the host than that of *L. plantarum* and/or *L. paracasei*, which are normally classified as free-living or nomadic (20, 21). Our data also suggest that our lactobacilli derive from a safe and docile environment, with virtually no potential to cause any significant harm, since all isolates are closely related to nonpathogenic and/or beneficial strains. Interestingly, the phylogenetic tree of the pediococcus isolates showed two different evolutionary clusters. *P. acidilactici* C5 from wild boar group 1 (zero TB prevalence) grouped in a cluster with other strains isolated from companion animals and healthy humans. The genome of C5 is also very similar to that of *P. acidilactici* ATCC 8042, a probiotic that has been extensively described (52–54). In contrast, *P. acidilactici* R91 from wild boar group 2 (high TB prevalence) shares the same cluster as strains originating from livestock and other outdoor environments. With regard to the enterococcus isolates, all of them were clustered with potentially pathogenic strains associated with severe clinical cases in animals (55). Both strains of E. faecalis (A1 and R8) are similar between them and also to the E. faecalis strain CVM N48037F, isolated from a sow (56).

In addition to the phenotypic and genotypic features that our LAB used to adapt to the host and environment, we also tested their antagonistic properties and ability to interact with the host’s immune cells and modulate innate immune responses. We first determined the potential of the isolates to antagonize bacteria that cause infectious diseases in wild boar, including M. bovis, E. coli, P. multocida, and L. monocytogenes. As reported in our previous work (23), lactobacilli from the TB-free estates (group 1) significantly inhibited M. bovis BCG, probably through the combination of multiple synergistic metabolites such as organic acids, hydrogen peroxide, ethanol, and unmodified two-peptide bacteriocins. Enterococci from the TB estates (group 2) showed a much lower reduction of BCG survival, which contradicts some authors who demonstrated the antimycobacterial effect of enterococci (57). Strikingly, the *P. acidilactici* strain (C5) from the TB-free group showed a much higher antimycobacterial activity than that of the *P. acidilactici* strain (R91) isolated from the estate with a high TB prevalence. One explanation for this significant divergence could be that C5 possess a larger number of genes, *Pox* and aldolase genes, that associate with the accumulation of hydrogen peroxide and lactic acid (16).

The antibacterial screening against the Gram-negative bacteria E. coli and P. multocida showed a species-specific response. The two E. faecalis strains (A1 and R8) showed the strongest antimicrobial activity against E. coli, and both contain clusters encoding a sactipeptide. This posttranslationally modified bacteriocin can outcompete Gram-negative bacteria under certain experimental conditions (58). An additional posttranslationally modified bacteriocin, lanthipeptide, was also identified in A1 and the *E. casseliflavus* isolate. However, the latter displayed a much weaker antimicrobial activity against E. coli, confirming a potential unique role of sactipeptides as antagonists to coliforms and other gut-related Gram negatives. Similarly, only the two *L. salivarius* strains (C2 and C12) displayed significant antimicrobial activity against the respiratory pathogen P. multocida. Previous studies have reported that bacteriocins and species that are phylogenetically close to *L. salivarius* may display inhibitory effects against *Pasteurellaceae* (21, 59–61). C12 carries a cluster involved in the production of an unmodified bacteriocin, and both C2 and C12 have the potential to produce multiple bacteriolysins, as we have previously reported (23). Bacteriolysins are extracellular enzymes that hydrolyze cell wall and membrane components of other bacteria that lack protective proteins against them (62, 63).

To complete the antimicrobial profile of the LAB isolates, we selected Listeria monocytogenes as a representative of Gram-positive pathogens in wild boar (64, 65). It is not surprising that the only strain displaying inhibition against L. monocytogenes was the pediocin producer *P. acidilactici* R91. Pediocin has been widely used as a food preservative due to its notable antilisterial activity (66–68). Furthermore, this bacteriocin is usually encoded by a 4-gene operon located in plasmids (69), suggesting its crucial involvement as a positive, evolutionary trait under unfavorable environments. The genome of the other *P. acidilactici* strain (C5), which derives from TB-free wild boar, does not possess any pediocin operon.

LAB are known to play an important role in the development of a proper immune system (2). However, little information is available regarding molecules and pathways involved in the cross talk between wildlife LAB and the host immune cells. Our final task was to explore the potential immunomodulatory properties of our wild boar isolates using porcine and human phagocytes as previously reported (23, 70). Except for *L. salivarius* C2, all lactobacilli interacted significantly with phagocytes, especially with monocytes. One possible mechanism that would explain this phagocytic response is the fact that isolates of *L. plantarum* and *L. paracasei* possess genes encoding two adhesins (*cna* and *sraP*) that have been reported to interact intimately with eukaryotic cells (71). However, we found no genetic determinants to support the evidence that *L. salivarius* C12 also interacts with monocytes. On the other hand, porcine blood cells challenged with pediococci and enterococci showed a poor phagocytic response, probably due to the ability of these bacteria to express evasion molecules such as the capsule polysaccharide YwqC (72). *E. casseliflavus* carries two more genes (*cap8A* and *capA*) associated with the biosynthesis of capsule molecules (73). Furthermore, the pediococcus isolates harbor no adhesin genes, and the genomes of the two E. faecalis strains contain a prolific set of genes that seem to deceive immune cells for subsequent cell colonization (3, 55, 74).

Interestingly, the activation of the NF-κB/IRF pathways in human macrophages exposed to the LAB isolates correlated positively with the phagocytic interaction observed with the porcine monocytes. In general, bacterial phagocytosis mediates protective innate immune responses via NF-κB or IRF (75, 76). On the one hand, *L. plantarum* and *L. paracasei* showed a very significant IRF profile, and both species are capable of binding to phagocytes as discussed above. In this respect, we know that Gram-positive bacteria normally activate the IRF pathway once they have been internalized and recognized by endosomal TLRs and cytosolic nucleic acid sensors (5, 77, 78). Likewise, the other phagocytic stimulator (*L. salivarius* C12) also triggered a significant macrophage response, but, in this case, it was dependent on NF-κB activation. On the other hand, although *L. salivarius* C2 and *P. acidilactici* C5 have no interaction with phagocytes, both strains were able to activate the NF-κB pathway. These results suggest an extracellular recognition by macrophages, including cell wall molecules such as LTA (2, 79), which seems to be abundant in *L. salivarius* C2. Another reason could be based on the evidence that the genome of C2 contains a gene coding for PrtP, a cell wall-anchored protease with immunomodulatory properties (2, 80). Similarly, *P. acidilactici* C5 has the potential to express stP, an S/T-rich protein that is involved in bacterial aggregation and innate immune modulation (2). Finally, it is worth emphasizing that all isolates from the wild boar group 2 (high TB prevalence) carry potential MAMPs, but none of them activated any of the pathways described. They did not interact with phagocytes either, highlighting the possibility that these bacteria have developed some deceit mechanisms to survive in very competitive, unfavorable environments.

### Conclusions.

Our study shows that viable LAB are useful indicators of the health status of the host and ecosystem from which they derive. We have seen that abundance of lactobacilli reflects a healthier wildlife population, whereas a profile defined by enterococci links with a more hostile environment. This leads us to believe that, with a simple microbiological analysis on fecal samples, it is possible to predict the health of wildlife animals by examining their most prominent symbiont LAB. To validate our hypothesis, it will be important to monitor additional microbiological parameters such as the use of next-generation sequencing to determine the microbiota composition of the collected fecal samples. Furthermore, we have observed that the phenotype and genotype of the LAB isolates correlate not only with their place of origin but also with specific antimicrobial and immunomodulatory properties. These properties, once again, seem to define host health, and to confirm their functional relationship with the genomic traits identified in this study, we propose future complementary studies, including defined gene and protein expression analyses.

The lactobacillus isolates, especially *L. plantarum* and *L. paracasei*, appear to stimulate protective responses against intracellular pathogens (5, 81). Both species show a very significant antimycobacterial profile, outcompete for phagocytic cells, and trigger antiviral IRF responses. The *L. salivarius* strains have also displayed some beneficial properties against potential bacterial infections due to their ability to inhibit P. multocida and boost NF-κB immune responses in macrophages. With regard to the pediococci, although we detected a lower immunomodulatory capacity than that of lactobacilli, preferably via NF-κB activation, one of the isolates revealed a very prominent activity against L. monocytogenes. This antilisterial activity is thought to be mediated by antimicrobial peptides (bacteriocins) such as pediocin (66, 67). Similarly, the enterococci seem to produce bacteriocins that antagonize E. coli. However, none of them interacted with immune cells. It is evident that LAB help the host to combat microbial infections, but in many different ways, protective mechanisms could be exploited to mitigate the spread of infectious diseases in wildlife ecosystems. Enterococci may pose a potential risk for animal or human health, but lactobacilli and pediococci are considered safe and used as probiotics worldwide. In this context, further probiotic interventions should be simultaneously complemented with epidemiological surveys monitoring the prevalence of pathogenic bacteria, such as E. coli, P. multocida, and L. monocytogenes, in wildlife ecosystems.

## MATERIALS AND METHODS

### Isolation, identification, and selection of LAB from wild boar feces.

Fecal samples were collected from at least 40 wild boar living in 4 different states in midwestern Spain, 10 animals per state. The estates were divided into two groups based on their TB epidemiological situation (see Table S1 in the supplemental material). Group 1 includes estates 1, 2, and 3 and represents a zero TB prevalence despite being located in regions with high TB risk for wild game animals. Group 2 comprises state 4 and distributes across a region with a historically high TB prevalence. The TB prevalence regions are officially classified according to Spanish Government legislation (RD 138/2020). LAB were isolated from the collected fecal samples and subjected to an antimicrobial screening as previously described (23). Briefly, rectal swabs were immersed in sterile peptone water (Oxoid) to prepare serial dilutions that were spread onto De Man, Rogosa, Sharpe (MRS; Oxoid) agar plates. After incubation at 37°C for 48 to 60 h under microaerophilic conditions, individual colonies were tested against Micrococcus luteus ATCC 4698 and Mycobacterium smegmatis mc^2^155 using replica plating (3). LAB isolates displaying antimicrobial activity were isolated and identified by Gram staining, catalase, and oxidase tests and 16S rRNA sequencing. We then selected the most representative and abundant genera from each of the estates to conduct further analyses. This LAB selection also considered different strains and species within the same state.

### Genome sequencing, assembly, and annotation.

DNA from 11 selected LAB isolates was extracted using the EZNA bacterial DNA kit (Omega Bio-Tek, USA), and the library preparation was carried out with the 250 Nextera XT library prep kit and genome sequencing using the Illumina MiSeq platform at MicrobesNG, University of Birmingham, United Kingdom. The quality of the generated reads was trimmed with Trimmomatic (82) and assessed using in-house scripts. Contigs were then generated and assembled from the paired-end reads using Shovill, version 1.0.4 (https://github.com/tseemann/shovill), with SPAdes 3.13.0 (83). The resulting genome assemblies were verified by *N*_50_ and *L*_50_ using Quast version 4.5 (84) and annotated with Prokka version 1.13 (85).

### Antibiotic susceptibility.

The sensitivity of the selected LAB isolates to antibiotics was quantified using the broth microdilution assay. As suggested by the EFSA Panel on Additives and Products or Substances used in Animal Feed (FEEDAP) (43), we used the following antibiotics: ampicillin, vancomycin, gentamicin, kanamycin, streptomycin, erythromycin, tetracycline, and chloramphenicol. Serial 2-fold dilutions of each of the antibiotics were tested against 2 × 10^5^ CFU/ml LAB in a Nunc-MicroWell 96-well plate (Thermo Scientific) for 18 h at 37°C. LAB growth was measured at 620 nm with a DTX 880 multimode detector microplate reader (Beckman Coulter) to calculate the MIC, which was defined as the lowest concentration of the antibiotic inhibiting LAB. Based on the MIC values and the microbiological cutoff values (mg/liter) defined by FEEDAP (43), we distinguished between resistant (R > *x* mg/liter) and susceptible (S ≤ *x* mg/liter) LAB.

### AMR and virulence markers.

We employed the Abricate version 0.9 (https://github.com/tseemann/abricate) to look at the LAB genomes for acquired AMR genes and putative virulence factors. Additional AMR genes were identified using the NCBI Bacterial AMR Reference Gene Database (https://www.ncbi.nlm.nih.gov/bioproject/PRJNA313047) and the two databases Resfinder (86) and Plasmifinder (87). The Virulence Factor Database platform (VFDB) (88) was also used to detect genes coding for virulence factors, such as proteinases, gelatinases, and biofilm formation proteins.

### LAB phylogenetic tree.

To determine the phylogenetic origin of our 11 LAB isolates, we carried out a genome comparison between them and identical LAB species isolated from the environment, raw or fermented food, and healthy or sick individuals, both human and animal. The publicly available genomes of *L. plantarum*, *L. salivarius*, *L. paracasei*, *Pediococcus* spp., E. faecalis, and *E. casseliflavus* were downloaded, along with their metadata, from the NCBI Genome Database using ncbi-genome-download version 0.2.9 (https://github.com/kblin/ncbi-genome-download). Due to the excessive number of genomes for *L. plantarum* and E. faecalis, we conducted a first comparative screening using purine-pyrimidine (FFPry) feature frequency profiling with a word length of 15 (89) to select the closest genome sequences. The genome comparison was then carried out using core genome single-nucleotide polymorphisms, as identified by ParSNP version 1.2 (90) and with settings previously described (91). Phylogenetic trees were generated with Figtree version 1.4.2 (http://tree.bio.ed.ac.uk/software/figtree/).

### Antimicrobial characterization.

The antimicrobial potential of our 11 LAB isolates was tested against Bacillus Calmette-Guerin (BCG) and 3 clinical isolates of Escherichia coli, Pasteurella multocida serotype B, and Listeria monocytogenes. BCG is a surrogate of M. bovis, the infectious source of bovine TB, while the clinical isolates are pathogenic bacteria associated with gut, respiratory, and reproductive infections. BCG was cultured in Middlebrook 7H9 (Difco) broth supplemented with 10% oleic acid-albumin-dextrose-catalase enrichment (OADC; Sigma-Aldrich), 0.05% Tween 80 (Sigma-Aldrich), and 0.2% glycerol at 37°C for 5 to 7 days. E. coli and P. multocida were grown in Mueller-Hinton (MH) broth (Oxoid) at 37°C for 24 h in an orbital shaker. L. monocytogenes and LAB were propagated without aeration at 37°C for 24 to 48 h in tryptone soy broth (TSB; Oxoid) and MRS broth, respectively. The methods of choice to monitor the antibacterial activity of the LAB isolates included (i) cocultures; (ii) broth microdilution assay; and (iii) spot-on-agar test, as previously described (3, 23, 70).

### Cocultures.

Each of the LAB were individually grown with a GFP-expressing BCG Pasteur strain (92) in enriched Mueller-Hinton (23) or with E. coli, P. multocida, or L. monocytogenes in TSB. As the selected broth media are not optimal for the growth of LAB, these were inoculated at a higher initial concentration (10^7^ CFU/ml) than with the indicator bacteria (10^5^ CFU/ml). With the exception of L. monocytogenes, cocultures were incubated with aeration at 37°C for 24 h to collect samples at different time points (0 and 24 h at least). The LAB-L. monocytogenes cocultures were incubated under static conditions. The survival rate of BCG was measured by GFP expression and indicated as fluorescence units (FU). The inhibitory effect on the clinical isolates was monitored by obtaining their corresponding numbers of CFU/ml and presented as log reductions. Numbers of CFU/ml were calculated from selective agar plates on which serial dilutions of the collected samples were spread. The selective agars were purchased from Oxoid and allowed for the enumeration of coliforms (violet red bile lactose), *Pasteurella* (*Pasteurella* selective medium), and *Listeria* (Palcam). Monocultures were also prepared and monitored as negative controls.

### Broth microdilution assay.

Cell-free supernatants of LAB were collected from 24-h MRS cultures after centrifugation at 5,000 rpm for 10 min and filtration through 0.22-μm-pore-size syringe filters (Branchia). Supernatants were serially 2-fold diluted and tested against a concentration of 10^7^ CFU/ml of BCG, E. coli, P. multocida, or L. monocytogenes. We followed the same protocol as that described for the MIC determination above but using the optimal broth for each of the indicator bacteria. Results were expressed as arbitrary units per milliliter (AU/ml) and defined as the reciprocal of the highest dilution showing clear growth inhibition of the indicator (93).

### Spot-on-agar test.

Spot-on-agar test is a very informative cell‐to‐cell antimicrobial test as previously reported by our group (94). Briefly, 5 μl of each LAB overnight cultures were spotted onto MH agar or tryptone soya agar (TSA; Oxoid) inoculated with 1% of a culture of BCG, E. coli, P. multocida, or L. monocytogenes at their late exponential phase. Plates were incubated at 37°C for 24 h (or 5 to 7 days for BCG) to evaluate growth inhibition zones. The following LAB strains were included as controls: *L. plantarum* WCFS-1, a strong acidifier (95); Pediococcus acidilactici PA1.0, an unmodified bacteriocin (pediocin) producer (69); Lactococcus lactis, a posttranslationally modified bacteriocin (nisin) producer; and Lactococcus lactis NZ9800, negative control of NZ9700 (96).

### Genes involved in the biosynthesis of antimicrobial compounds.

Genomes were uploaded onto BAGEL4 (http://bagel4.molgenrug.nl/), a very reliable web server that enables rapid identification of gene clusters associated with the production of bacteriocins (97). Bacteriocins are posttranslationally modified (or unmodified) antimicrobial peptides that are ribosomally synthesized and originate from clusters containing genes encoding the bacteriocin precursor and proteins involved in modifications, processing, extracellular transport, and immunity (3). Additionally, we used the genome annotations to carry out a very extensive search for genes and pathways from which potential antimicrobial secondary metabolites may derive. Target genes comprised those that code for enzymes associated with hydrogen peroxide (H_2_O_2_) production, including NADH oxidases (*nox*), pyruvate oxidase (*pox*), lactate dehydrogenase-oxidase (*ldh*-*lox*), and NADH-dependent flavin reductases (98). H_2_O_2_ scavengers such as NADH peroxidases were also monitored. Furthermore, we also characterized the two main glycolytic pathways of LAB, the Embden-Meyerhof pathway (EMP) and the phosphoketolase pathway (PKP), based on the presence of fructose-6-phosphate aldolase and phosphoketolase (PK), respectively (8). Both pathways are crucial for the biosynthesis of organic acids and other potent antimicrobials. Homofermentative LAB only contain the aldolase, converting carbohydrates into lactate using EMP. In contrast, heterofermentative LAB possess both aldolase and PK, resulting in the production of lactate, acetate, ethanol, and carbon dioxide via PKP (99).

### Phagocytosis assay.

Whole blood was collected from pigs and treated accordingly as previously described (23). Blood was mixed with PKH2-labeled LAB, incubated at 37°C for 30 min, and lysed with red blood cell solution (BioLegend). FACS sorting was then carried out using the flow cytometer BD FACS Celesta to distinguish the main blood cell populations (3). This differentiation is based on forward (FSC) and side (SSC) scatter and corresponds to the size and granularity of blood cells (lymphocytes versus phagocytes). Simultaneously, the fluorescein isothiocyanate (FITC) channel was also used to measure the GFP levels in blood cells that bind (e.g., lymphocytes) and/or phagocytose (e.g., phagocytes including monocytes and polymorphonuclear leukocytes [PMNs], such as neutrophils). The resulting SSC/GFP plot illustrated the quantification of LAB intake by phagocytes.

### Response of macrophages to LAB.

We used the monocyte cell lines of THP-1-IFIT-1-GLuc (REF) and THP1-Lucia NF-κB (Invivogen) to monitor the activation of the transcription factors IRF and NF-κB upon exposure to LAB, as previously described (3). IFIT1-GLuc cells secrete Gaussia luciferase (GLuc) under the control of the promoter of the interferon-induced protein with tetratricopeptide repeat IFIT1, while those of NF-κB-GLuc do the same but under the control of the NF-κB promoter. Both cell lines were grown in Roswell Park Memorial Institute (RPMI; Sigma-Aldrich) 1640 medium with 15% fetal calf serum (FCS; Seralaf) and 1% penicillin-streptomycin (Pen-Strep; Life Technologies) at 37°C in an atmosphere of 5% CO_2_. We treated the cells with 20 ng/ml phorbol 12-myristate 13-acetate (PMA) to differentiate them to macrophages that were subsequently challenged with LAB at a ratio of 20 viable bacteria per macrophage in RPMI with 2% FCS for 2 h. After the challenge, we changed the medium and left the macrophages in RPMI containing 2% FCS and 1% Pen-Strep for 20 h. The activation of IFIT1 and NF-κB was then measured in a Clariostar plate reader (BMG Biotech) using coelenterazine (NanoLigh Technology) at 2 μg/ml. Activation was calculated as a fold increase of luciferase activity over the measurements recorded for unchallenged macrophages.

### Immunomodulation markers.

To identify markers associated with host innate immune responses, we followed the same genome procedure as that described for the AMR and virulence markers. We paid special attention to proteins involved in mechanisms of evasion, adhesion, and colonization using Abricate on the VFDB database. Furthermore, the genome annotations were explored to identify LAB ligands that normally interact with PRRs, such as TLRs or NOD-like receptors, including exopolysaccharides (EPS), pilus (fimbrial) precursors, lipoteichoic acid (LTA), wall teichoic acid (WTA), and membrane lipoproteins (2, 100, 101).

### Statistical analysis.

Statistical analysis was performed using GraphPad Prism version 8.00 for Windows (GraphPad Software, La Jolla, CA). Data are means ± standard deviations (SD) and represent at least three biological replicates. Differences between isolates were analyzed by one-way analysis of variance (ANOVA) followed by Fisher’s least significant difference (LSD) test.

### Data availability.

Sequencing reads, genome assemblies, and metadata have been uploaded to GenBank under BioProject no. PRJNA544176 and PRJNA544254.
