# Supplementary material for: Wildlife Symbiotic Bacteria Are Indicators of the Health Status of the Host and Its Ecosystem
Source: Appl Environ Microbiol. 2022 Jan 11;88(1):e01385-21. doi: 10.1128/AEM.01385-21 (PMC8752132; doi:10.1128/AEM.01385-21)
Supplement: Supplemental file 1 — Fig. S1 to S5, Table S1. Download AEM.01385-21-s0001.pdf, PDF file, 0.8 MB [file aem.01385-21-s0001.pdf]

Supplemental figure S1. Phylogenetic tree of *L. plantarum* isolates. All of *L. plantarum* isolates are related to strains isolated from fermented food or probiotic related products. EML1 and SA3 are almost identical and both show similarities with isolate C1. Origins of strains are represented in the tree as follows: wild boar isolates (pink), animal (purple), fermented or raw food (red), human (green), dairy (blue) and environment or plants (yellow).

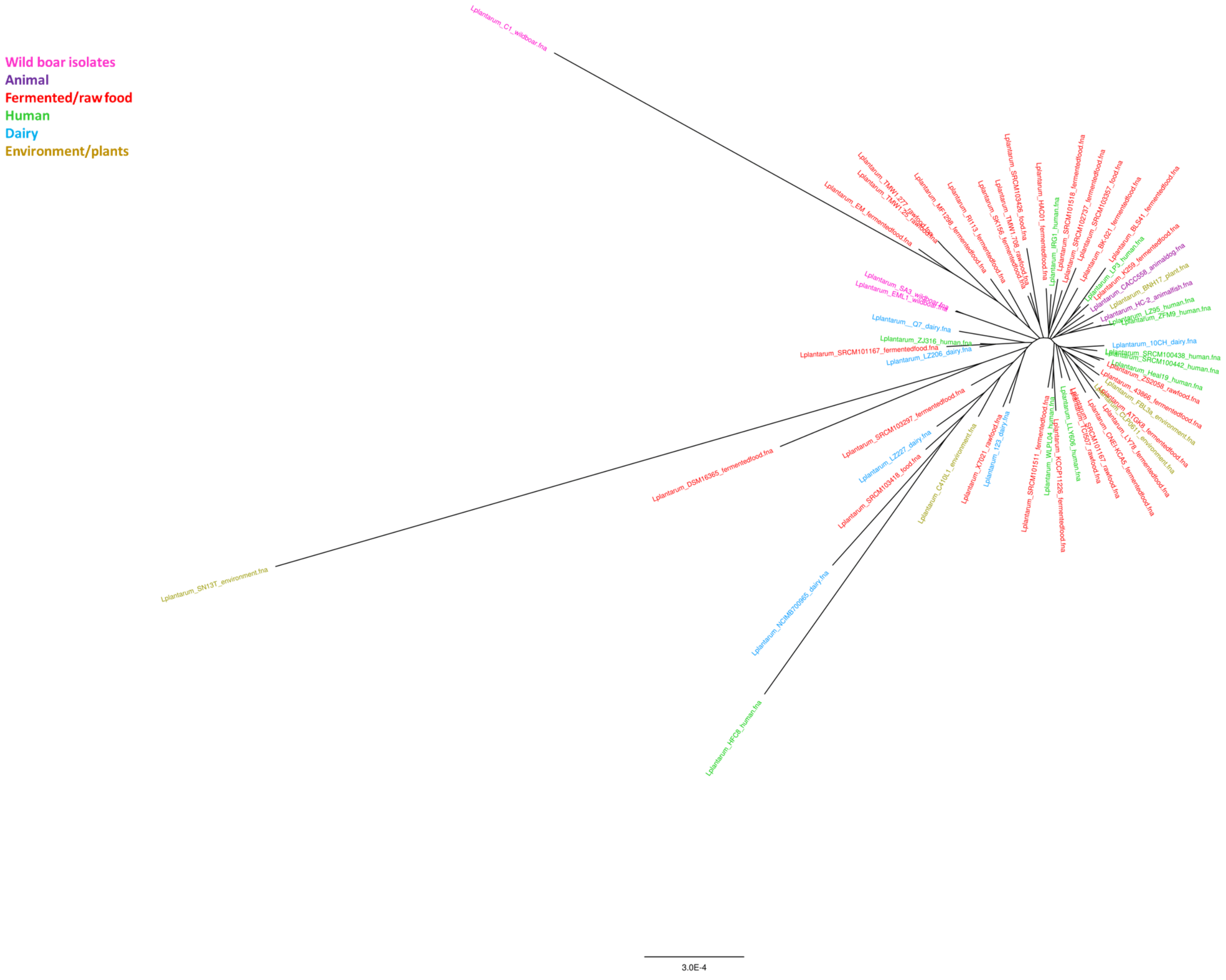

Supplemental figure S2. Phylogenetic tree of *L. paracasei* isolates. *L. paracasei* SA5 is close to strains derived from food such as dairy products. Origins of strains are represented in the tree as follows: wild boar isolates (pink), animal (purple), fermented or raw food (red), drink (dark red), human (green), dairy (blue) and environment or plants (yellow).

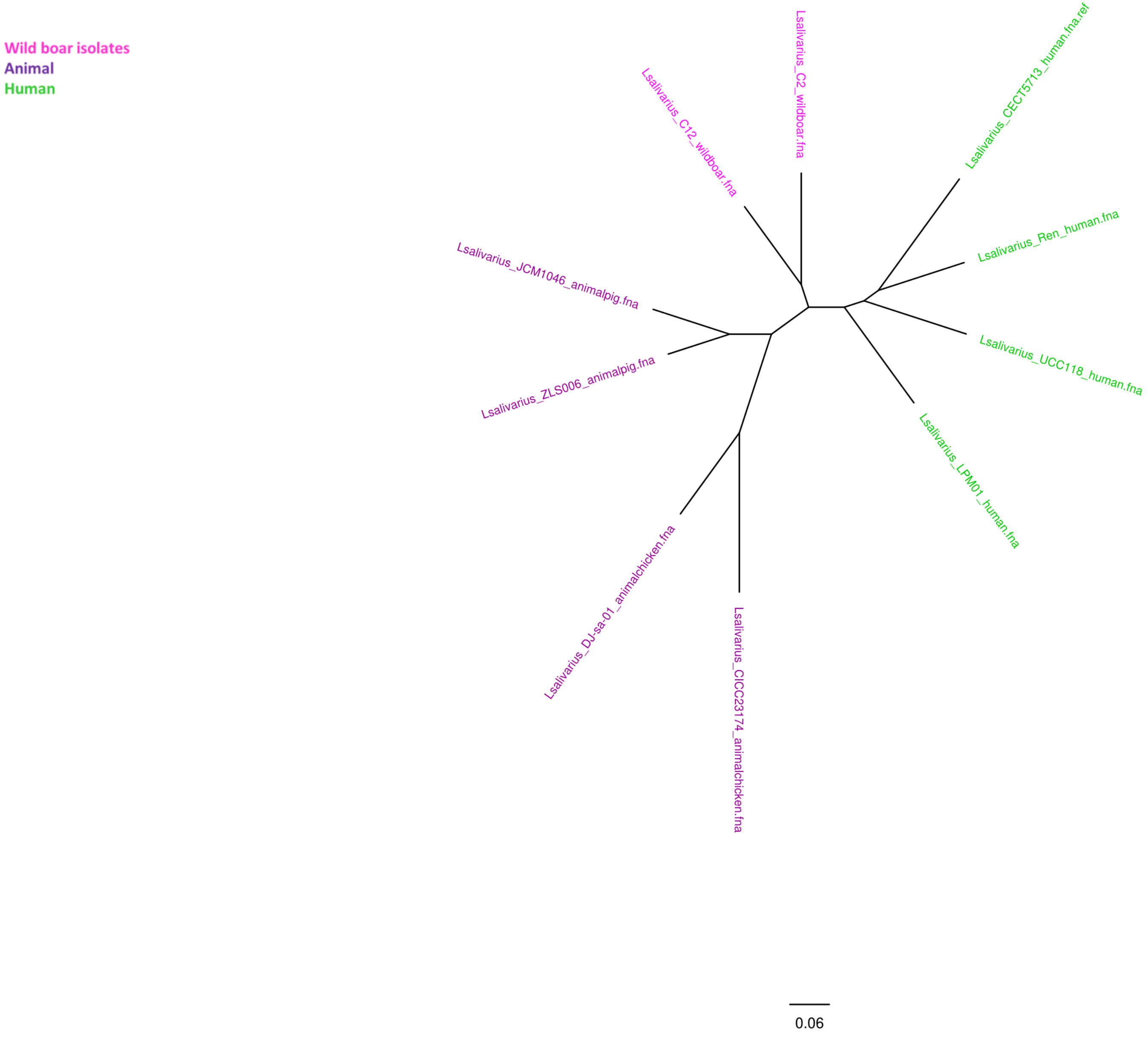

Supplemental figure S3. Phylogenetic tree of *L. salivarius* isolates. The *L. salivarius* isolates C2 and C12 locate between two groups representing strains from pigs and humans. Origins of strains are represented in the tree as follows: wild boar isolates (pink), animal (purple), and human (green).

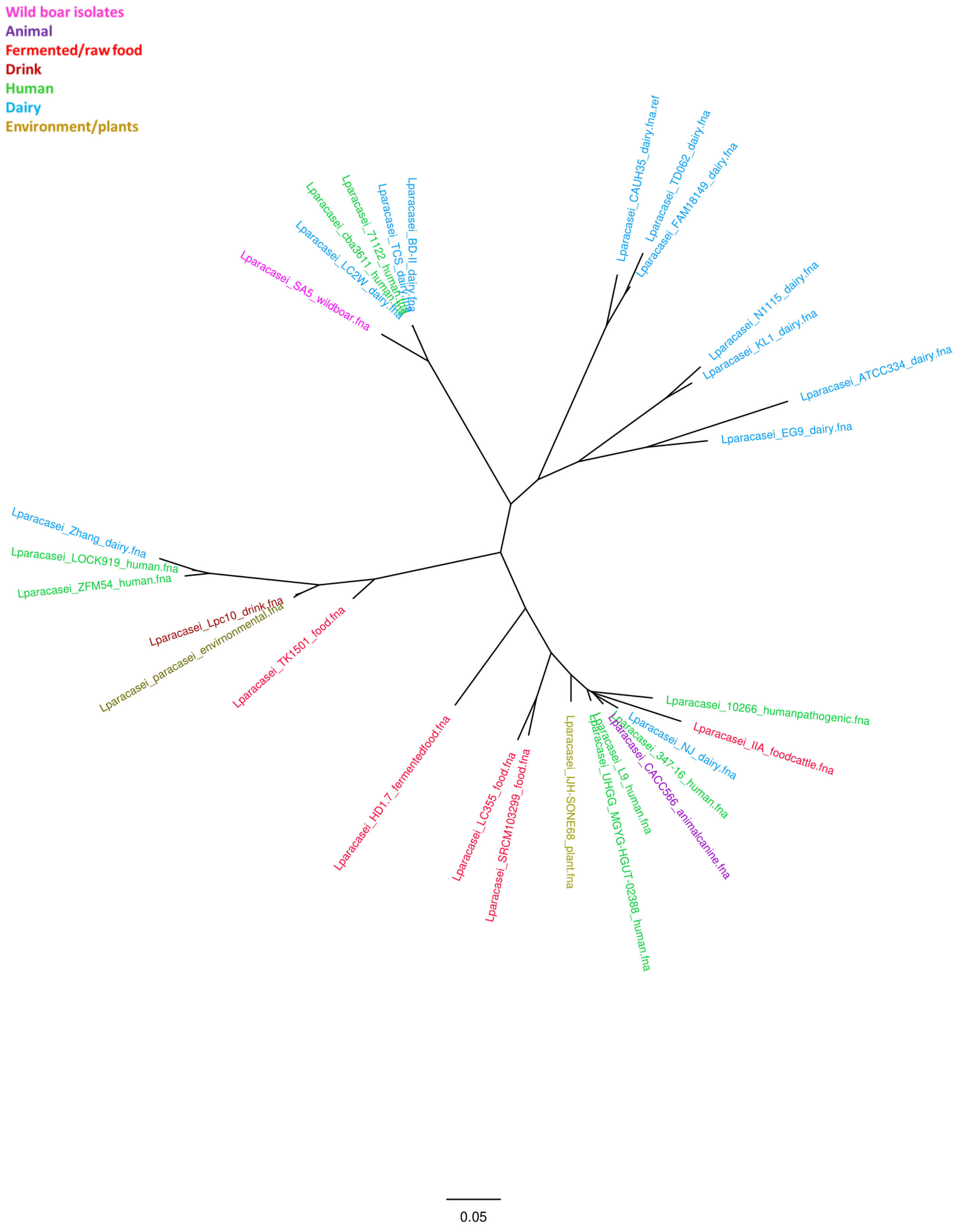

Supplemental figure S4. Phylogenetic tree of *P. acidilactici* isolates. StrainC5 belongs to a group of isolates from fermented food, humans and pet animals (dog), whereas R91 associates with a group of strains that derive from food (fermented and dairy) and farm animals (chicken and pig). Origins of strains are represented in the tree as follows: wild boar isolates (pink), animal (purple), fermented or raw food (red), human (green) and dairy (blue)

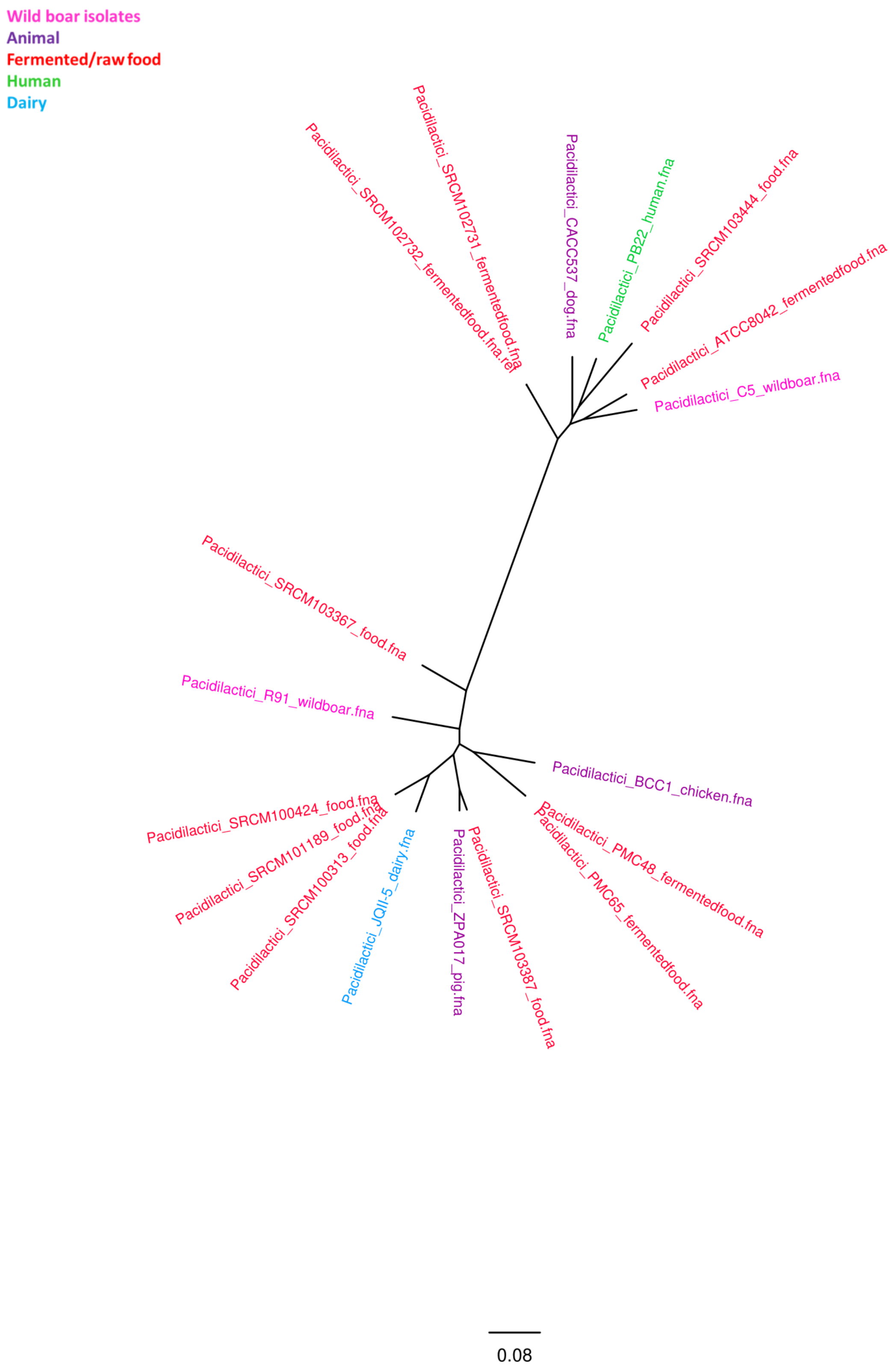

Supplemental figure S5. Phylogenetic tree of *E. faecalis* isolates. *E. faecalis* A1 and R8 are very close to pathogenic strains isolated from pigs. Origins of strains are represented in the tree as follows: wild boar isolates (pink), animal (purple), fermented or raw food (red), human (green), dairy (blue) and environment or plants (yellow).

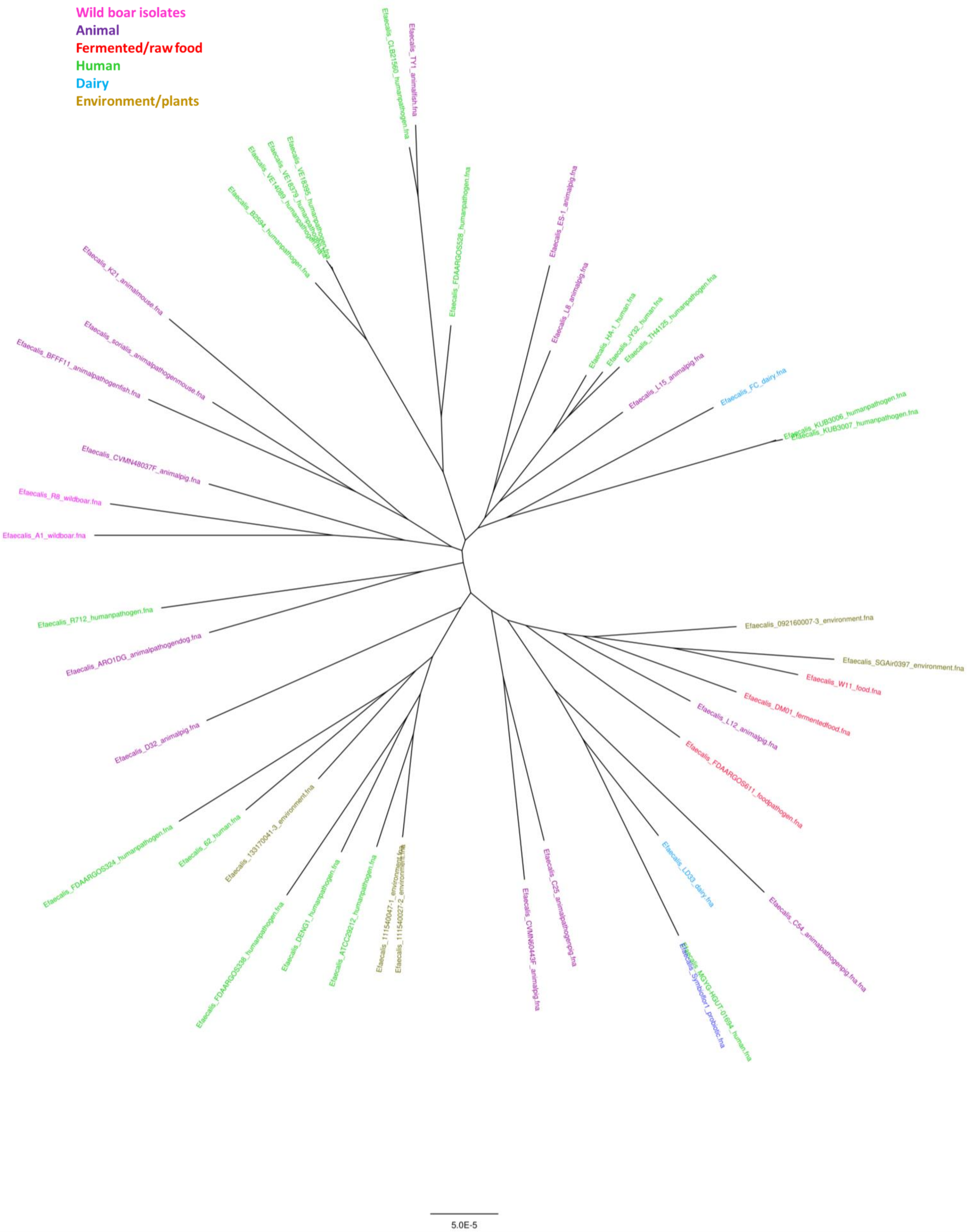

Supplemental table 1. Supplemental information about estates included in the study.

	Estates			
	**1**	**2**	**3**	**4**
**Postal code (Spain)**	16541	06650	13100	10695
**Area** (Ha)	800	500	2500	4900
**Density** (wild boar / Km^2)	45	75	35	5-10
**Feeding**	Wild boar fodder and cereals	Wild boar fodder and maize	Wild boar fodder, cereals and milk serum	No supplementation
**Ration**	*Ad libitum*	0.7 kg / animal	*Ad libitum*	-
**Cohabitat with other ungulates**	*Capreolus capreolus*	*Cervus elaphus and Ovis musimo)*	*Cervus elaphus, Ovis musimo and Capreolus capreolus*	*Cervus elaphus*
**TB gross lesions prevalence (%)**	0	0	0	20
**Other diseases**	Not detected	*Chlamydia* Mal rojo *(Erysipelothrix rhusiopathiae)*	*Salmonella cholerasuis, Escherichia coli, Pasteurella multocida* type B	-
